# Understanding disciplinary vocabularies using a full-text enabled domain-independent term extraction approach

**DOI:** 10.1371/journal.pone.0187762

**Published:** 2017-11-29

**Authors:** Erjia Yan, Jake Williams, Zheng Chen

**Affiliations:** College of Computing and Informatics, Drexel University, Philadelphia, Pennsylvania, United States of America; KU Leuven, BELGIUM

## Abstract

Publication metadata help deliver rich analyses of scholarly communication. However, research concepts and ideas are more effectively expressed through unstructured fields such as full texts. Thus, the goals of this paper are to employ a full-text enabled method to extract terms relevant to disciplinary vocabularies, and through them, to understand the relationships between disciplines. This paper uses an efficient, domain-independent term extraction method to extract disciplinary vocabularies from a large multidisciplinary corpus of *PLoS ONE* publications. It finds a power-law pattern in the frequency distributions of terms present in each discipline, indicating a semantic richness potentially sufficient for further study and advanced analysis. The salient relationships amongst these vocabularies become apparent in application of a principal component analysis. For example, Mathematics and Computer and Information Sciences were found to have similar vocabulary use patterns along with Engineering and Physics; while Chemistry and the Social Sciences were found to exhibit contrasting vocabulary use patterns along with the Earth Sciences and Chemistry. These results have implications to studies of scholarly communication as scholars attempt to identify the epistemological cultures of disciplines, and as a full text-based methodology could lead to machine learning applications in the automated classification of scholarly work according to disciplinary vocabularies.

## Introduction

The bibliometric community has used scientific publications as an effective instrument to study scholarly communication. Traditionally, bibliometric indicators were employed to assess research impacts [1–3]. Recent advances in bibliometrics have benefited from the use of network and statistical approaches to map science [4–6] and identify author communities [7–10]. Publication metadata, such as authors, journals, and references, were primarily used as the unit of analysis in these prior endeavors. The use of a more content-rich component—full-texts—was largely absent. Consequently, we made great efforts in examining research metadata but not research contents.

The composition of the research landscape is evolving—data, particularly scientific data, are increasing becoming open and accessible. The increased access to data not only provides more efficient means of analyses, but also entails a paradigmatic shift in modes of inquiry as scientists now can form diverse teams surrounded by data and conduct data-intensive research. The success of this transformation requires the use of new methods to extract more granular and content-rich information from large publication data. This need is within the realm of information extraction since computational linguists have developed methods to identify terms that can be used to describe domain-specific concepts from texts. While modern natural language processing techniques have yielded satisfying results on recall and precision, they were primarily employed with the objective of retrieval, as opposed to understanding. Accordingly, systematic approaches are lacking on how to utilize these methods to understand the latent meanings of the texts of scientific publications and how to use them to address questions on scholarly communication.

Thus, the objectives of this paper are two-fold. First, it is motivated to develop a term weighting-based method to extract content-rich terms from full texts. These terms can be broadly perceived as expressions in texts that convey information about the research-relevant aspects of publications, such as methods, theories, and concepts. Second, it uses the extracted terms to compare and contrast disciplines’ vocabularies—these vocabularies are important signifiers of disciplinary discourse patterns and can be used to reveal the epistemological differences in disciplinary cultures, as Hyland [11] argued that “writing…[o]n the contrary, it helps to create those disciplines”. The newly developed term extraction method allows us to examine the epistemological differences in a heretofore unattained extent, which complements the scholarship of the language aspect of disciplinarity studies that were largely confined to analyze samples of articles [12], dissertations [13], textbooks [14], and book reviews [15].

The paper provides insights into disciplinary vocabulary patterns and reveals scholarly communication at a new contextualized level. Conducting content-rich disciplinarity studies has the readily apparent advantage of gaining concrete and fine-grained perceptions of how different scientific concepts are embedded and relate to each other. It also helps us obtain an in-depth understanding of the production and dissemination patterns of scientific knowledge, innovations, and influences. By automatically extracting large and disciplinarily specific vocabularies, the satisfaction of this work’s goals also opens avenues for large-scale applications through algorithms that may use these rich lexica as feature inputs for machine learning.

## Literature review

Recent years have witnessed a growing interest in term extraction. The term extraction task is concerned with two concepts, unithood and termhood. Unithood deals with the syntactics of terms and is formally defined as “the degree of strength or stability of syntagmatic combinations of collections” [16]. Termhood focuses on the semantic representation of terms or in Kageura and Umino’s words “the degree that a linguistic unit is related to…domain-specific concepts” [16]. Scholars have employed both linguistic and statistical methods to extract terms with unithood and termhood in mind from a variety of textual genres, such as email correspondences [17], scientific publications [18], and the Web [19]. Applications range from bioinformatics to studies of political parties [20]—giving rise to a new research area called named entity recognition and classification (NERC) [21]. Named entities encompass a variety of actors and artifacts such as people, locations, organizations, and biomedical entities. Three types of NERC methods are present: unsupervised, semi-supervised, and supervised. They are introduced in this section.

Unsupervised methods use lexical resources (e.g., WordNet or Web queries) [22, 23] and lexical patterns (e.g., the “such as” pattern) [21] to extract named entities. This approach has advantage because it provided a high-level validity of unithood. As for termhood, scholars have differentiated the weight of noun phrases according to certain measures, such as an entropy-based index [24], a context-based term weighing method [25], or a uniqueness-based indicator that compares word frequencies between scientific and non-scientific corpora [18, 26]. The idea behind the uniqueness-based indicator (a.k.a “weirdness”) is that terms in scientific and non-scientific corpora have disparate frequencies, making it is possible to use standard non-scientific corpora, such as the British National Corpus, to filter technical terms from scientific corpora [18, 26]. Reports of high precision [18] suggest potential for this method in applications to domain-independent corpora.

Semi-supervised methods typically use a bootstrapping technique. This technique recursively learns the contextual patterns of a small number of seed terms and uses the learned patterns to select new terms. Bootstrapping is an “effective, interpretable” [27] method and has performed well at extracting domain-dependent terms relating to terrorism [28], law [29], and medicine [30]. Supervised methods primarily include maximum entropy models [31], support vector machines [32], decision trees [33], hidden Markov models [34], and conditional random fields [35, 36]. These methods perform well, and extract named entities using labeled data [37]. However, requirements for large training data with entity-class associations result in a high complexity of O(D×R), where D is the number of documents and R is the number of relations [19]. Thus, developing Web-scale, domain-independent methods is recognized as a priority in the NERC community. Milestone events in this vein of research include the KnowItAll [19] and TextRunner systems [38]. KnowItAll was the first published domain-independent system, according to Etzioni, Banko [19]. The performance of the system, however, was impeded by the high volumes of Web query requests and the system-wide adjustment every time a new relation was added. TextRunner resolved these scalability issues and is seen as a “fully implemented” open information extraction (OIE) system [19]. It supported the discovery of new entity-class associations and reduced the complexity to O(D).

## Methods and data

### Term extraction

This paper employs a new term extraction method for full-text scientific copra developed in our prior research [39]. The method processes texts through StanfordNLP for lemmatization and part-of-speech (POS) tagging. Original texts, lemmas and POS tags then go through the POS matching procedure to identify possible terms, i.e., candidates, which can be a word or a phrase of multiple words. Candidates are further scored by our term extraction algorithm, which will be discussed in the following paragraphs. After POS tagging, each word is associated with a POS tag (Penn Treebank: [40]). POS tag sequences are matched by POS patterns. We chose the following pattern to match candidate terms: (({JJ}|({NN}[{VBG}|{VBN}]?)|{CD})+{IN})?({JJ}|({NN}[{VBG}|{VBN}]?)|{CD})*{NN}, where JJ denotes adjectives, NN denotes nouns, VBG denotes verbs in gerund or present participle, VBN denotes verbs in past participle, CD denotes cardinal numbers, and IN denotes prepositions. This pattern is selected based on our heuristic observations that valid terms are a combination of nouns, prepositions, adjectives, and verbs in present or past participles. Table 1 shows several examples of terms extracted by this pattern.

**Table 1:** Examples of extracted terms using the defined POS pattern.

POS tag sequence	Extracted terms
JJ NN NN	mesenchymal stem cell
NN JJ NN	mouse embryonic fibroblast
NN IN NN	mutation in gene
VBG NN	cold-seeking response
NN VBN NN	hiv-2 uninfected individual
NN JJ NN	brand new innovation
NN IN NN	people with history

This pattern is capable of including as many POS structures as possible; however, because it is designed to maximize recall, the candidate terms are considerably noisy (see the last two examples in Table 1). To select valid terms from these candidates, formula (1) is adopted to score each candidate.

$$score_{d}(x)\;=\;C_{d}(x)\times W_{1}(x)\times W_{2}(x)$$

In formula (1), *C*_*d*_(*x*) is the C-Value formula given by Frantzi, Ananiadou [25], and *W*_1_(*x*), *W*_2_(*x*) are our weighting functions. C-Value relies on features such as term lengths and document-level frequencies. These features, however, cannot effectively distinguish scientific concepts from ordinary expressions. Thus, we propose an extension to include two types of frequency lists in scoring to improve performance.

We define the type I frequency list as a list of word frequencies acquired from a non-scientific corpus, for example, news articles and novels. In this work, we obtain a type I list from the Project Gutenberg eBooks repository for our method (https://en.wiktionary.org/wiki/Wiktionary:Frequency_lists/PG/2006/04/1-10000). We then employed the sigmoid function to weigh down candidate terms that contain high-ranking words in the type I reference list.

$$W_{1}(x)\;=\;\frac{\sum_{\omega \in x}\left(\frac{1}{1+e^{\frac{r_{0}-r_{\omega }}{s_{1}}}}-\alpha_{1}\right)}{\left(1-\alpha_{1})|x\right|}$$

where *ω* is a word in the term *x*, and *r*_*ω*_ is the rank of *ω* in the type I frequency list. If *ω* is not in the list, we let *r*_*ω*_ = +∞. $g(\omega )\;=\;\frac{1}{1+e^{\frac{r_{0}-r_{\omega }}{s}}}$ is a sigmoid function. *r*_0_ is a constant that controls when the function takes value 0.5. For example, if *r*_0_ = 4000, then *g*(*ω*) = 0.5 if *r*_*ω*_ = 4000, meaning a word ranked 4000 in the frequency list will be mapped to a sigmoid value 0.5. The other constant *s*_1_ controls the function’s rate of increasing with respect to *r*_*ω*_. An increase of *s*_1_ will make the sigmoid increase slower, and a decrease of *s*_1_ will make the sigmoid increase faster. $\alpha_{1}\;=\;\frac{1}{1+e^{\frac{r_{0}}{s_{1}}}}$ which is used to normalize *W*_1_(*x*) between 0 and 1. This reflects the idea of uniqueness of scientific terms—top words in daily language tend not to appear in them.

We define a type II frequency list as a list of word frequencies from a scientific terminology dictionary. For some words, such as “protein” and “behavior”, there presence in a candidate boosts the likelihood of them being a scientific term. Moreover, we observe that the position of a word in a term can also be used to determine the likelihood. For example, the word “central” is unlikely to appear at the end of any valid term and the word “theory” tends to tail valid terms. A second weighting function is designed as:

$$W_{2}(x)\;=\;1+\frac{\sum_{\omega \in x}\left(\left(\frac{1}{1+e^{\frac{f_{0}-f_{\omega }}{s_{2}}}}-\alpha_{2})\times (1-\beta |p(\omega )-\overset{-}{p}(\omega )|\right)\right)}{\left(1-\alpha_{2})|x\right|}$$

where *ω* is a word in the term *x*, and *f*_*ω*_ is the frequency of *ω* in the type II frequency list. If *ω* is not in the list, we let *f*_*ω*_ = 0. Similar to *W*_1_(*x*), $h(\omega )\;=\;\frac{1}{1+e^{\frac{f_{0}-f_{\omega }}{t}}}$ is also a sigmoid function. s_2_ is a slope parameter as described before for *W*_1_(*x*). Also we let $\alpha_{2}\;=\;\frac{1}{1+e^{\frac{f_{0}}{s_{2}}}}$ for the purpose of normalization. We define the normalized position (NP) of a word *ω* in *x* as its zero-based position in *x* divided by |*x*|−1, the length of *x* minus one. For example, in term “central limit theorem”, “central” is at position 0 and is of NP 0, “limit” is at position 1 and is of NP “0.5”, and “theorem” is at position 2 and of NP “1”. $\overset{-}{p}(\omega )$ is the average normalized position of word *ω* of all terms that generate the type II frequency list. As a result $\left|p(\omega )-\overset{-}{p}(\omega )\right|$ is a deviation of *ω*’s position in *x* from *ω*’s average position in the terminology dictionary, or simply the position disagreement, and $1-|p(\omega )-\overset{-}{p}(\omega )|$ is the position agreement. In addition, *β* ∈ [0,1] is a weighting parameter that controls on what level position disagreement affects *W*_2_(*x*).

A comprehensive type II reference list is relatively less available than a type I list. We recommend the use of acronyms to build up a type II list. Currently, the type II list is constructed from the literature itself based on recognized acronyms. About 10 thousand acronyms were recognized from our *PLoS ONE* corpus by using a simply rule-based approach (i.e., the existence of title case capitalization and parentheses) and we used these acronyms to build a type II list. It is worth noting that this term extraction method gives a higher score to words on the type II list and can find terms outside of this list. The benefit of using a type II list is that it can be considered comprehensive or well-rounded with respect to the literature corpus we analyze, and it reduces the method’s dependence on external resources.

Our method is advantageous because it does not rely on any corpus-level features such like document frequency, and thus it is able to process publications without first processing the whole corpus. We showed in [39] that our term extraction method outperformed the state-of-the-art, C-Value method and summarize this key finding in Table 2.

**Table 2:** Evaluation results.

	C-Value	W_1_ Weighted	W_1_ W_2_ Weighted
Precision	0.8170	0.9390	0.9520
Recall	0.4230	0.4670	0.4990

Precision in Table 2 refers to the ratio of the number of technical relevant terms among the top 20 extracted terms over all top 20 terms. Three human coders conducted the evaluation over 50 documents; a term is considered as a non-technical relevant term when a consensus was reached among all three coders. Recall refers to keyword recall; we used keywords as the gold standard when evaluating recall of the three methods. We see from the evaluation results that our method improved precision by 15% when *score*_*d*_(*x*) = *c*_*d*_(*x*) × *W*_1_(*x*) was used and by 17% when *score*_*d*_(*x*) = *c*_*d*_(*x*) × *W*_1_(*x*) × *W*_2_(*x*) was used. In regards to recall, our method (*score*_*d*_(*x*) = *c*_*d*_(*x*) × *W*_1_(*x*) × *W*_2_(*x*)) boosted the recall by 18% compared with the C-Value method.

### Data

The dataset used in this paper contains 52,981 *PLoS ONE* articles published between 2006 and 2015. The access point to the corpus is provided by *PLoS ONE* (http://www.plosone.org/google/index.html) and it is freely accessible to the public. For each article in the dataset, we applied the designed term extraction method and selected top 40 terms based on *score*_*d*_. In total, we collected 532,725 unique terms from the dataset.

To examine disciplinary vocabularies, papers in the dataset need to be grouped into appropriate disciplines. *PLoS* has a classification scheme that assigns a paper to two or more research areas. We noticed that some research areas are quite similar and thus reclassified the research areas into 12 broader disciplines based on research similarities (Table 3).

**Table 3:** Reclassification of *PLoS* subjects.

	Reclassified disciplines	Original disciplines
1	Agriculture	Agriculture
2	Biology	Biology; Biology and life sciences; Biology and life sciences; Veterinary science
3	Chemistry	Chemistry
4	Computer and Information Sciences	Computer science; Computer and information sciences
5	Earth Sciences	Earth sciences
6	Ecology and Environmental Sciences	Ecology and environmental sciences
7	Engineering	Engineering and technology; Engineering; Materials science
8	Mathematics	Mathematics
9	Medicine and Health Sciences	Medicine and health sciences; Medicine
10	Physics	Physics; Astronomical sciences; Physical sciences
11	Research and Analysis Methods	Research and analysis methods
12	Social Sciences	Social sciences; Social and behavioral sciences; People and places; Science policy

Multi-counting was adopted in that a paper is counted in each discipline it was assigned into (see S1 Table for the summary of papers with multiple-subject assignments). For instance, a paper assigned into Biology and life sciences and Chemistry in the original scheme was counted once in Biology and once in Chemistry in the reclassified scheme. The advantage of this counting method is that it avoids counting a paper in an arbitrary discipline [41]. The caveat, however, is that it blurred the disciplinary boundaries and we should be cautious when interpreting results on interdisciplinarity. Another limitation of the employed classification scheme is that despite *PLoS ONE*’s multidisciplinary scope, this journal has a more extensive coverage on biomedical-related topics. This has raised a dilemma to us: on the one hand, we are interested to include in our analysis more representative domain-specific journals; on the other hand, most domain-specific journals outside biomedicine are not open access and thus would hinder the study’s reproducibility. As more journals are having open access options, we see the use of open access, domain-specific journals to examine disciplinary vocabularies as a future research direction.

## Results

### An overview of disciplinary vocabularies

Table 4 shows the number of publications, number of unique terms, and terms per paper for each discipline.

**Table 4:** Descriptive statistics of the 12 disciplines.

Disciplines	No. of publications	No. of unique terms	Terms per paper (tpp)
Agriculture	2,047	42,005	20.52
Biology	41,136	497,492	12.09
Chemistry	12,530	195,853	15.63
Computer and Information Sciences	8,356	67,296	8.05
Earth Sciences	1,676	29,971	17.88
Ecology and Environmental Sciences	7,638	117,838	15.43
Engineering	2,866	102,327	35.70
Mathematics	2,793	53,372	19.11
Medicine and Health Sciences	25,068	368,896	14.72
Physics	4,773	87,349	18.30
Research and Analysis Methods	3,089	33,764	10.93
Social Sciences	7,514	67,328	8.96

While Biology and Medicine had the highest numbers of unique terms, Engineering possessed the highest terms per paper (tpp = 35.70). Other disciplines that resulted in high terms per paper include Agriculture (tpp = 20.52), Mathematics (tpp = 19.11), and Physics (tpp = 18.30). The result suggests that these disciplines tended to use nomenclatures more frequently in texts. Meanwhile, Computer and Information Sciences (tpp = 8.05) and Social Sciences (tpp = 8.96) yielded the lowest terms per paper. The result that Computer and Information Sciences had fewer terms per paper comes as a surprise, because this field is often seen as technology-driven. Thus, it is expected that it pertains to more frequent uses of technical terms—similar to the case of Engineering. It is possible that given PLoS ONE’s multidisciplinary nature, papers accepted by it in the area of computer science are primarily on the application track and used fewer technical terms than those on the theory and method tracks that are often published in domain-specific journals and conference proceedings.

To gain an understanding of the basic research themes of each discipline, we show top terms of each discipline. We first calculated the ratio of the occurrence of a term in one discipline against its occurrences in all 12 disciplines. This ratio helps bring terms that have a more salient association with certain disciplines. We then set up a threshold of 1,000 documents, meaning that a term needs to occur in at least 1,000 documents to be considered as top terms. Finally, we ranked the ratios in the descending order, with results shown in Table 5.

**Table 5:** Top 10 terms of each discipline.

	**Agriculture**	**Biology**	**Chemistry**	**Comp**	**Earth**	**Ecology**
1	seedling	chromatin	SDS-PAGE	algorithm	ecosystem	biodiversity
2	molecular marker	chromatin immunoprecipitation	mutant protein	node	environmental variable	predation
3	transcriptome	wnt	phospholipid	dataset	biomass	habitat
4	cm	zebrafish	cysteine	database	biodiversity	ecosystem
5	nutrient	histone	ion	functional annotation	nutrient	environmental variable
6	genetic diversity	dorsal	fusion protein	simulation	habitat	biomass
7	functional annotation	transcriptional regulator	recombinant protein	matrix	predation	conservation
8	genome sequence	chromosome	protease	equation	China	nutrient
9	biomass	phylogenetic analysis	tyrosine	dynamics	gradient	mammal
10	cellular component	recombination	proteasome	parameter	cm	genetic diversity
	**Engineering**	**Math**	**Medicine**	**Physics**	**Research**	**Social Sciences**
1	seedling	equation	CD8 T cell	equation	heterogeneity	SD
2	laser	algorithm	CD4	dynamics	follow-up	variable
3	algorithm	dynamics	CD4 T cell	orientation	meta-analysis	evaluation
4	electron	node	T cell	voltage	injection	sensitivity
5	ph	parameter	follow-up	ligand	questionnaire	ANOVA
6	fusion protein	dataset	HIV	microtubule	diagnosis	questionnaire
7	voltage	simulation	morbidity	simulation	diabetes mellitus	impact
8	ml	heterogeneity	IL-10	ion	consensus	meta-analysis
9	gel	regression coefficient	vaccination	node	metabolic syndrome	respondent
10	PCR amplification	matrix	diagnosis	radiation	OR	feedback

Table 5 shows that the designed term extraction method is able to identify both words and phrases from texts. These words and phrases are capable of depicting the general research themes of each discipline. For instance, Computer Science examines algorithms and networks; Earth Sciences is centered with environmental studies; Ecology focuses on biodiversity and conservation, Medicine discusses T cells and vaccinations; and Social Sciences seem to cover topics on both qualitative and quantitative research methods.

### Term distributions

**Fig 1:**
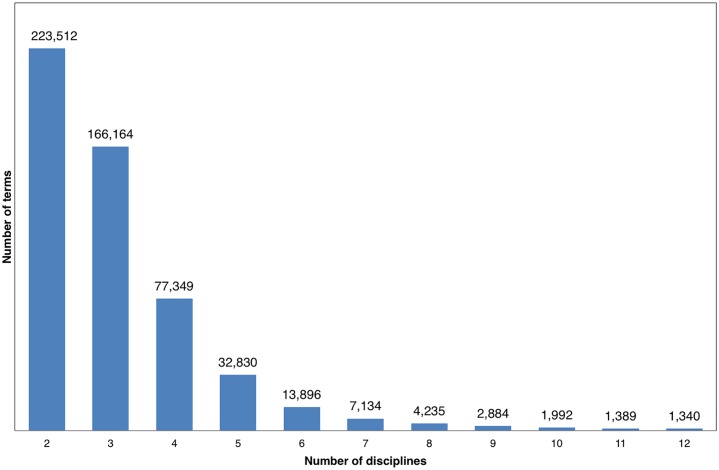
The distribution of terms in disciplines.

**Fig 2:**
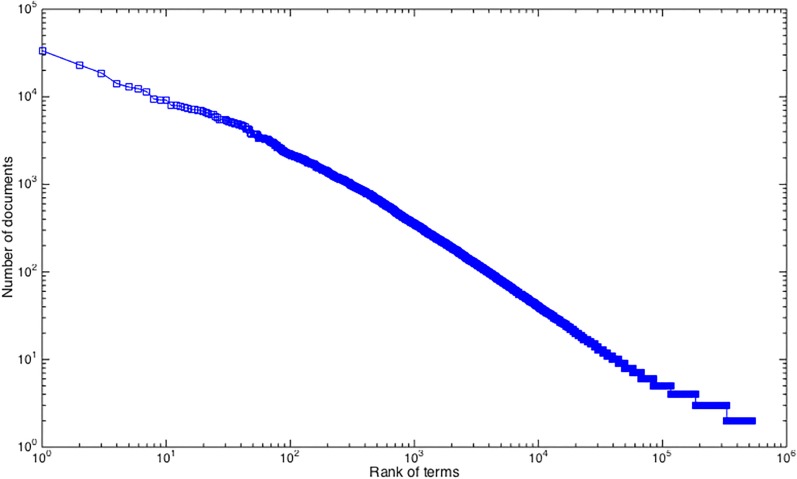
The rank-frequency distribution of terms in documents.

**Fig 3:**
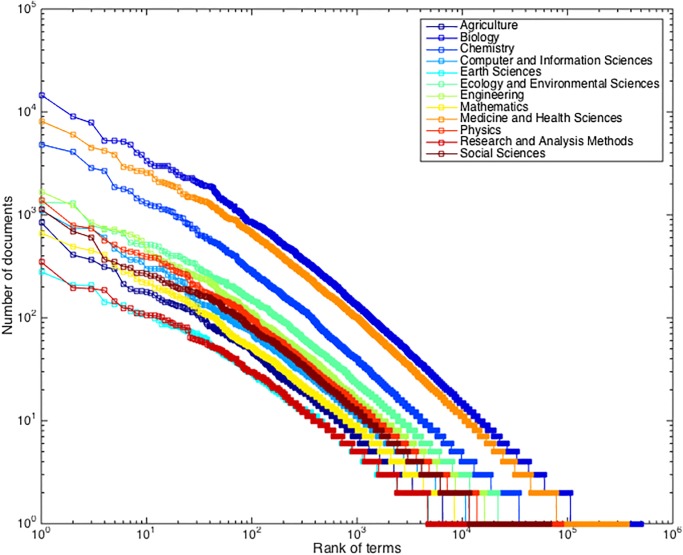
The rank-frequency distribution of terms in documents for 12 disciplines.

In this subsection, we show the distributions of terms over disciplines (Fig 1) and documents (Figs 2 and 3). Fig 1 illustrates the numbers of terms that occurred in one or more disciplines. Note no term was found only in one discipline because papers were assigned to at least two disciplines in *PLoS ONE*.

More than 40% of the terms (223,512) were associated with two disciplines and as the number of disciplines increases, the number of terms declines. There are 1,340 terms (0.25%) that occurred in all 12 disciplines. Among these, the following terms are the ones with the highest document-level occurrence (document-level occurrences in parentheses): gene (33,594), protein (23,143), antibody (18,709), USA (14,180), enzyme (12,855), and apoptosis (12,356). Meanwhile, for terms that only occurred in two disciplines, those with the highest document-level occurrence are CD27 (51), CD8 t-cell response (48), tetramer staining (27), pulmonary macrophage (26), centrosome amplification (24), and hematopoietic stem cell transplantation (24). We can see that terms in the latter group are more granular.

Fig 2 shows the distribution of terms over documents in a log-log scale. The y-axis shows the number of documents a term occurs and the x-axis shows the order of terms, from the term that occurs in the highest number of documents to the terms that occur in just two documents.

A power law pattern is visible in Fig 2 because of the linear distribution between the number of documents and the rank of terms in the log-log scale with base 10. This pattern shows that while most terms only occurred in a small number of documents, some terms occurred in most documents [42], such as gene, protein, antibody, USA, enzyme, apoptosis, and genome that occurred in more than 10,000 documents. In the meantime, there are 202,536 terms that only occurred in two documents.

We now zoom in to examine the term document distribution in each discipline. Fig 3 shows the distributions of terms over documents for all 12 disciplines in a log-log scale with the same axis compositions.

Distributions in Fig 3 share a similar pattern with Fig 2 in that there is a power law relationship between the number of documents and the rank of terms. Curves’ slopes are consistent, exhibiting a parallel form among the curves. The difference, however, is the intercept on the y-axis: Biology, for instance, had the largest number of terms and documents and thus has the highest intercepting value; Earth Sciences, on the other hand, has the lowest intercepting value.

### Cross-discipline vocabulary similarities

**Fig 4:**
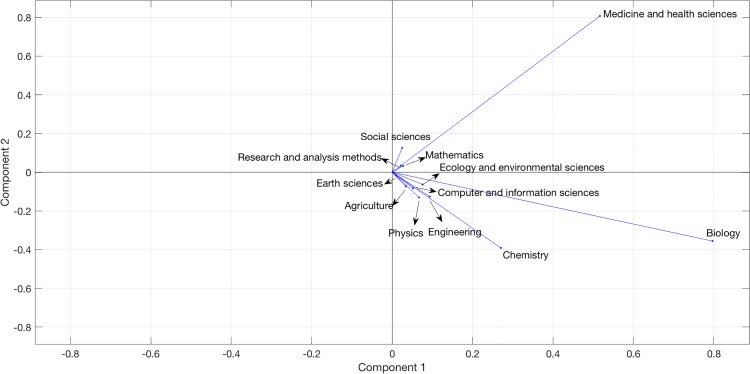
The plot of the first two components of the principal component analysis.

We employed a principal component analysis (PCA) to measure disciplines’ similarities based on the vocabularies disciplines used in scientific publications. The PCA was applied to a 12 by 532,725 discipline-term matrix where each cell contains the number of occurrences of a term in a discipline. The largest two components in PCA accounted for 97.8% of the total variance (95.4% and 2.4% respectively) and they are visualized in Fig 4.

**Fig 5:**
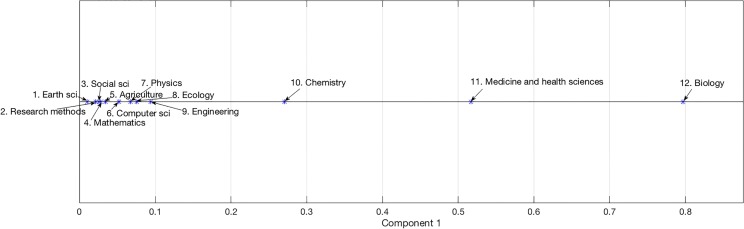
The plot of the first component of the principal component analysis.

Several observations can be made regarding the PCA plot. First, all domains are located in the first and fourth quadrants because the loadings for each domain in the first component is non-negative. Second, Mathematics, Medicine and Health Sciences, Research and analysis methods, and Social Sciences are in the first quadrant, while the other domains are in the fourth quadrant. Third, Ecology, Earth Sciences, and Agriculture are closely located in the fourth quadrant. Also closely located in the fourth quadrant are Computer and Information Sciences, Engineering, and Physics. Fourth, Biology, Chemistry, and Medicine are seemingly far part on the plane, but because the first component accounted for more than 95% of the total variances, the three domains are in effect closely located when projecting them on the x-axis. This close relationship can be seen more clearly in Fig 5 where only the first component is visualized. Numbers before the domain names are their ranks projected on the x-axis in that Earth Sciences (0.01, 0) is the closest to the origin and Biology (0.80, 0) the furthest.

**Fig 6:**
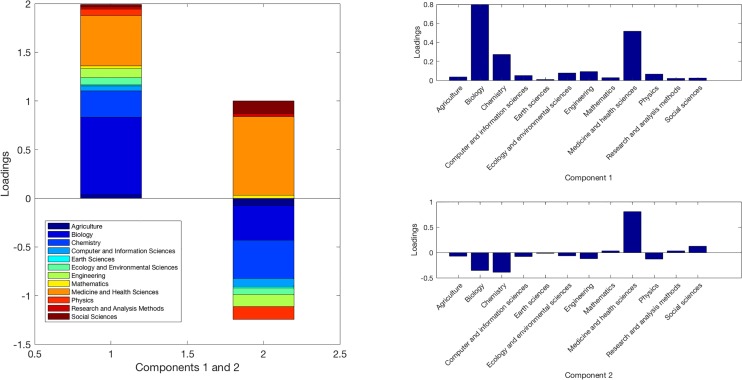
Stacked loadings of the first two components.

To show the loadings of the first two components, we provide a stacked bar chart (Fig 6). For consistency, the same color coding of Fig 3 is used for Fig 6.

Biology has the largest loading in the first component, followed by Medicine, and Chemistry. Meanwhile, Earth Sciences, Research and Analysis Methods, and Social Sciences have the smallest loadings in the first component. The results indicate the dominant role of biochemical terms in the first component, which is not surprising given that a large percentage of *PLoS ONE* publications are classified under Biology, Chemistry, or Medicine. Besides these three domains, Ecology, Engineering, and Physics also made noticeable contributions to the first component. The first component, therefore, is led by biochemistry and physical sciences. While all domains’ loadings are non-negative in the first component, only four domains’ loadings are non-negative in the second component (Mathematics, Medicine, Research and Analysis Methods, and Social Sciences) and others’ have negative loadings. The fact that Medicine has the largest loading while Biology and Chemistry have the largest negative loadings show that the latter two domains are more closely related through their use of terms from Medicine. This difference, however, is very subtle, due to the small variances the second component contributed.

## Discussion

When we projected the 12 domains on the first component, we obtained a discipline similarity chain: Earth Sciences->Research and Analysis Methods->Social Sciences->Mathematics->Agriculture->Computer and Information Sciences->Physics->Ecology and Environmental Sciences->Engineering->Chemistry->Medicine and Health Sciences->Biology. We compare the chain with the “consensus map” in [43], which was created by merging 20 existing science maps and identifying the general proximity patterns of disciplines: starting from mathematics, there are “physics, physical chemistry, engineering, chemistry, earth sciences, biology, biochemistry, infectious diseases, medicine, health services, brain research, psychology, humanities, social sciences, and computer science”.

A few similarities can be identified between the term occurrence map (i.e., the chain) and the consensus map: in the consensus map, social science and computer science are collocated and in the term occurrence map, Social Sciences and Computer and Information Sciences are also closely located to each other, separated by two domains Mathematics and Agriculture. Physics, engineering, and chemistry are collocated in the consensus map while Physics, Ecology, Engineering, and Chemistry are collocated in the term occurrence map. In the consensus map, biology and a few medical science domains are collocated while in the term occurrence map, Medicine and Biology are collocated. A few differences can be found, which are understood through two main factors: first, the PCA plot obtained from this study is based on 12 broad knowledge domains whereas previous science maps were based on more-granular units, such as the 27 Scopus major subject areas [44], 220 Web of Science subject categories [45], thousands of journals [5, 46], or millions of documents [47]. The more-granular units provided the possibility of using richer dimensions to depict the similarity between scientific fields. Second, previous science maps were created upon article- or journal-level co-citation relationships, whereas the plot in Fig 4 was created through discipline-level term occurrence relationships.

This paper found several disciplinary vocabulary use patterns. While Engineering tended to use nomenclatures more frequently in texts, Computer and Information Sciences and Social Sciences had the lowest numbers of terms per paper. In addition, based on a principal component analysis, this paper found that Mathematics and Computer and Information Sciences had a similar vocabulary use pattern, as did the pairing of Engineering and Physics. According to Carnap [48], disciplines in each of the abovementioned pairs pertained to “a very narrow and homogeneous class of terms of the physical thing-language”. Meanwhile, Chemistry and Social Sciences exhibited contrasting vocabulary use patterns, as did Earth Sciences and Chemistry. The results may have implications to studies of scholarly communication as scholars attempt to identify the epistemological cultures of disciplines [11], find disciplinary knowledge paths [49, 50], promote interdisciplinary research and collaborations [51], and design effective indicators to assess research outputs [52].

The term extraction method developed in this paper complements current work in co-word analysis. Co-word analysis can be very useful in portraying the cognitive space of a variety of disciplines [53, 54]. It is predicated upon a few assumptions [55]: keywords symbolize “non-trivial relationship between their referents” and indexers assign reliable keywords to refer to scientific concepts. In reality, however, due to the so-called “indexer effect” [56, 57], these assumptions may not be fully met, and the performance of co-word analyses may be impaired. To alleviate this tension, there is a growing interest of using title or abstract words instead of keywords [58]. The choice over title and abstract words may grant a “more direct access to the view of authors” [55] and may create a richer content to extract scientific concepts and ideas. The presented term extraction method can provide further refinement to co-word analysis from two aspects: first, this method is capable of extracting noun phrases—this holds clear advantage over single word-based extraction because many scientific concepts contain more than one word. Second, because of its weighting mechanism, the presented method can be applied to full texts that are richer in content and the extracted terms of a paper are weighed and ranked and the most distinctive terms can be used in co-word analysis.

## Conclusion

This paper employed an efficient, domain-independent term extraction method to extract disciplinary vocabularies from a large multidisciplinary corpus of *PLoS ONE* publications. The employed method can effectively extract content-rich terms from unstructured bibliometric fields such as full texts used in this study. Extracted terms can help researchers and practitioners contextualize findings and make sense of bibliometric indicators and numbers. Examinations of the extracted terms can help reveal the scholarly communication at a new granular level and address questions on the provenance, diffusion, coevolution, trend, and impact of knowledge at a much improved extent and depth. Analyzing and modeling content-rich terms also complements the state-of-the-art data infrastructure that orients towards network analysis of publications [6, 59–61]. In addition, this paper also found a power law pattern of the distribution of terms over documents: a small number of terms occurred in most documents while most others only occurred in a limited number of documents. This distribution pattern was also present for documents in each discipline, indicating the existence of a large and natural quantity of discipline-specific terms sufficient to application of statistical analyses machine learning algorithms.

Developing effective term extraction methods applicable to the full texts of scientific literature is the first step of a greater effort to enable content-aware bibliometric research. Next step will likely include the design and application of automated methods to induct taxonomies to organize extracted terms, in addition to the development of statistical methods and machine learning algorithms that may leverage extracted terms to automatically classify the ever-growing scientific literature. Thus, in the context of increasing interdisciplinarity, this effort should have long-term benefits to knowledge management and information retrieval. Future work will also include the employment of statistical- and network-based methods to understand the lifecycle of innovations as codified by content-rich terms.

## Supporting information

S1 Table Paper assignment table. (DOCX)

S1 File Term extraction algorithm. (RAR)
