# Supplementary material for: Understanding disciplinary vocabularies using a full-text enabled domain-independent term extraction approach
Source: PLoS One. 2017 Nov 29;12(11):e0187762. doi: 10.1371/journal.pone.0187762 (PMC5706669; doi:10.1371/journal.pone.0187762)
Supplement: S1 Table — (DOCX) [file pone.0187762.s001.docx]

|  | Agriculture | Biology | Chemistry | Comp | Earth | Ecology | Engineering | Math | Medicine | Physics | Research | Social sciences |
| --- | --- | --- | --- | --- | --- | --- | --- | --- | --- | --- | --- | --- |
| Agriculture | 0 | 1859 | 502 | 57 | 158 | 751 | 655 | 46 | 624 | 67 | 50 | 109 |
| Biology | 1859 | 0 | 11230 | 2480 | 1143 | 5714 | 4437 | 1614 | 22654 | 3650 | 1125 | 2296 |
| Chemistry | 502 | 11230 | 0 | 757 | 330 | 999 | 1926 | 273 | 6683 | 2118 | 400 | 180 |
| Comp | 57 | 2480 | 757 | 0 | 119 | 283 | 766 | 806 | 1134 | 672 | 164 | 478 |
| Earth | 158 | 1143 | 330 | 119 | 0 | 1010 | 228 | 68 | 287 | 119 | 26 | 184 |
| Ecology | 751 | 5714 | 999 | 283 | 1010 | 0 | 606 | 271 | 2463 | 264 | 98 | 419 |
| Engineering | 655 | 4437 | 1926 | 766 | 228 | 606 | 0 | 361 | 2443 | 932 | 196 | 306 |
| Math | 46 | 1614 | 273 | 806 | 68 | 271 | 361 | 0 | 1327 | 739 | 212 | 445 |
| Medicine | 624 | 22654 | 6683 | 1134 | 287 | 2463 | 2443 | 1327 | 0 | 1792 | 1046 | 2296 |
| Physics | 67 | 3650 | 2118 | 672 | 119 | 264 | 932 | 739 | 1792 | 0 | 327 | 427 |
| Research | 50 | 1125 | 400 | 164 | 26 | 98 | 196 | 212 | 1046 | 327 | 0 | 131 |
| Social sciences | 109 | 2296 | 180 | 478 | 184 | 419 | 306 | 445 | 2296 | 427 | 131 | 0 |

Table S1. Number of papers that were assigned to both the column and row subjects
